# Structural Differentiation of Graphs Using Hosoya-Based Indices

**DOI:** 10.1371/journal.pone.0102459

**Published:** 2014-07-14

**Authors:** Matthias Dehmer, Abbe Mowshowitz, Yongtang Shi

**Affiliations:** 1 Department of Computer Science, Universität der Bundeswehr München, Neubiberg, Germany; 2 Institute for Bioinformatics and Translational Research, UMIT, Hall in Tyrol, Austria; 3 Department of Computer Science, The City College of New York (CUNY), New York, New York, United States of America; 4 Center for Combinatorics and LPMC-TJKLC, Nankai University, Tianjin, China; 5 College of Computer and Control Engineering, Nankai University, Tianjin, China; University of Maribor, Slovenia

## Abstract

In this paper, we introduce the Hosoya-Spectral indices and the Hosoya information content of a graph. The first measure combines structural information captured by partial Hosoya polynomials and graph spectra. The latter is a graph entropy measure which is based on blocks consisting of vertices with the same partial Hosoya polynomial. We evaluate the discrimination power of these quantities by interpreting numerical results.

## Introduction

Structural differentiation entails the classification of graphs according to structural features captured by quantitative measures, see, e.g., [1]–[5]. One way to demonstrate a classification procedure is to apply a measure (or index) to a special class of graphs and show that the measure discriminates between non-isomorphic graphs with high probability. A prominent example is the Balaban 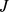 index [6]–[8] which is highly discriminating on chemical graphs. However, this index has limitations as shown by Dehmer et al. [2] by means of a statistical analysis of the performance of the 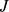 and other indices on an exhaustively generated set of graphs without structural constraints, see [2]. This analysis shows that the discrimination power (also called uniqueness [2]) of graph measures depends on the underlying class of graphs [2].

This paper is an investigation of the discriminating power of structural indices based on the zeros of partial Hosoya polynomials and graph spectra. Also, we introduce and evaluate the *Hosoya information content* of a graph. To position this investigation we begin with a survey of literature dealing with eigenvalues and entropy-based measures of graphs. Classical results in the theory of graph spectra are due to Cvetković et al. [9]. The main concern of this theory is to explore structural properties of graphs and complex networks captured by graph spectra [10]. More recent results have been presented and surveyed by Chung [11] and Cioab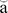 [12]. Interdisciplinary applications of graph spectra, e.g., the analysis of biological networks and web graphs can be found in [10], [13]. Various graph measures incorporating eigenvalues have been discussed by Randić et al. [14] and Dehmer et al. [15]. One example of a measure is defined as the sum of the moduli of non-zero eigenvalues of the adjacency matrix of a graph; another is given by graph entropies based on the eigenvalues of matrices associated with a graph [14]–[16]. Yet another well-known measure is the Estrada index [17]–[20] which has been explored in bioinformatics, mathematical chemistry and applied mathematics. A more recent review of this quantity is due to Gutman et al. [21]. Variants of this measures using other matrices have been discussed by Li et al. [22]. A related measure is the so-called energy of a graph is an important quantity defined in relation to the eigenvalues of matrices associated with a graph, see [23]–[25]. Extremal properties of graph energy have been studied by [23]–[25]. A recent book on graph energy summarizing classical and new results is [26]. Inequalities for eigenvalue-based graph measures have been discussed in [12]. Elphick and Wocjan [27] analyzed a novel spectral measure for determining network irregularity [27].

Graph entropy measures have been explored extensively in various disciplines. Rashevsky and Mowshowitz did seminal work when developing the first graph entropy measures based on vertex orbits [28], [29]. Körner introduced a graph entropy measure that has been used in information theory [30]. Bonchev et al. developed the magnitude-based information indices and various others based on graph invariants such as vertex degrees and distances in graphs [31]–[34]. Also, Bonchev et al. [1] proposed an information index for graphs which is based on the Hosoya graph decomposition. However, this information index (using Hosoya index 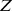 [35] to define the probabilities of the induced partition) is quite different from the one we introduce here in section ‘Hosoya-based Indices’. Many other graph entropy measures can be found in [36]–[38]. To study results towards the Hosoya polynomial, we refer to [39], [40].

In an earlier paper [3], we explored the discrimination power of measures (see section ‘Hosoya-based Indices’) that are based on the moduli of the zeros of the partial Hosoya polynomial. The main contribution of this paper is to define the *Hosoya-Spectral indices* combining structural information captured by partial Hosoya polynomials with graph spectra. Also, we examine the discrimination power of these indices and of the Hosoya information content of a graph. We discuss and compare the numerical results with the earlier ones produced in [3]. Further we elaborate on the usage of these measures as highly discriminating graph invariants.

## Methods and Results

### Hosoya-Based Indices

In this section, we reproduce the graph indices based on partial Hosoya polynomials, see [3]. As outlined in [3], the partial Hosoya polynomial of a vertex 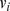 in the graph 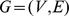 is given by [41], [42]

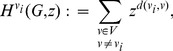
(1)where 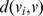 is the distance (i.e., length of a shortest path) between the vertices 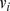 and 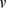. Solving the equation
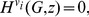
(2)yields the complex zeros 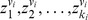 which are not equal to zero. We infer 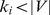 by applying the well-known fundamental theorem of Algebra [43], [44] stating that a complex polynomial
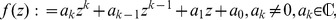
(3)with degree 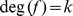 has 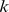 complex zeros.

Also in [3], Dehmer et al. introduced the following indices:
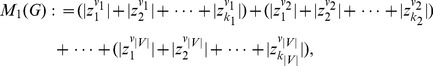
(4)

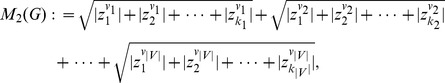
(5)and



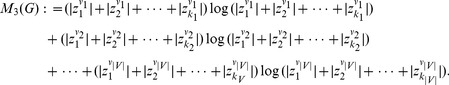
(6)Here, 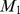 is the sum of the sums of the moduli of all partial Hosoya polynomials 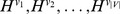. 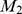 is the sum of the square roots of the sums of the moduli of all partial Hosoya polynomials. 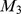 represents an entropy-like measure taking the sums of the moduli of all partial Hosoya polynomials into account.

Spectra of graphs have been investigated extensively [9], [12]. As already mentioned, well-known spectral based indices are the Estrada index [18]–[20] and various forms of graph energy due to Gutman, see [23]–[25]. Since Hosoya polynomials and graph spectra capture different aspects of graph structure, we propose to combine the two in one index. So, let 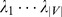 be the eigenvalues of 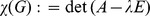; 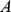 the adjacency matrix of 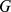. The *Hosoya-Spectral indices* 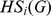 are defined as follows:
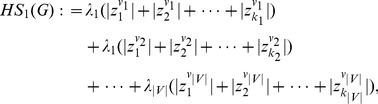
(7)

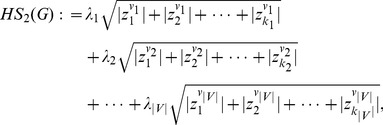
(8)

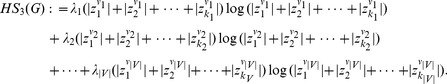
(9)


Inspired by studying information-theoretic complexity measures [1], [28], [31]–[33], [45], we define the *Hosoya information content* of a graph 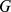. Let 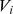 for 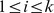 be the set of all vertices in 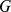 with the 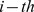 of 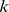 partial Hosoya polynomials of the vertices of 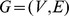; 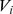 is then the 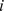-th block in a partition of 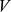. The *Hosoya information content* of 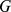 is defined by
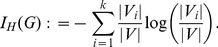
(10)


In the next section, we evaluate the discrimination power of this new measure and the Hosoya-Spectral indices on exhaustively generated graphs.

### Numerical Results

As in [3], we present the numerical values resulting from the evaluation of the discrimination power of the Hosoya-Spectral indices and the Hosoya information content. In order to do so, we use the same graph classes in order to make direct comparisons [3]; 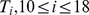 are the sets of all non-isomorphic trees with 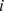 vertices. 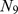 is the set of all non-isomorphic graphs with 9 vertices, see [2].

To evaluate the discrimination power quantitatively, we use the same measures as in [3]: ndv stands for the number of non-distinguishable graphs according to the values of the indices. From this, we also compute 
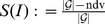
 where 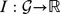, see [46].

As in [3], the measures 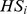 are fully unique on 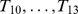, see Table 1. We obtain the same result by applying the Hosoya-Spectral indices to 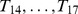, see Table 2. See also [3]. Moreover, the Hosoya-Spectral indices can discriminate the tree class 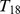 uniquely (see Table 3). Note that the earlier defined measures 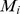 (see Equation 4–6) [3] produced the first degeneracies on the set 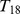.

**Table 1:** Exhaustively generated sets of non-isomorphic trees: 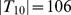, 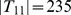, 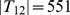, 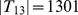.

	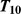		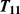		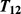		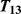	
Measure	ndv	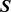	ndv	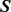	ndv	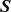	ndv	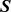
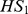	0	1,000000	0	1,000000	0	1,000000	0	1,000000
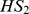	0	1,000000	0	1,000000	0	1,000000	0	1,000000
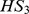	0	1,000000	0	1,000000	0	1,000000	0	1,000000
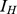	76	0,283018	201	0,144680	499	0,094373	1237	0,049192

**Table 2:** Exhaustively generated sets of non-isomorphic trees: 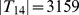, 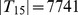, 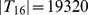, 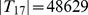.

	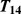		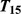		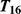		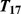	
Measure	ndv	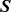	ndv	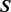	ndv	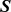	ndv	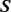
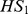	0	1,000000	0	1,000000	0	1,000000	0	1,000000
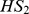	0	1,000000	0	1,000000	0	1,000000	0	1,000000
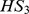	0	1,000000	0	1,000000	0	1,000000	0	1,000000
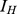	3067	0,029123	7637	0,013434	19178	0,007349	48629	0,003783

**Table 3:** Exhaustively generated sets of non-isomorphic trees and graphs: 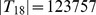, 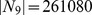.

	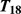		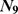	
Measure	ndv	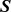	ndv	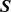
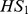	0	1,000000	24	0,999908
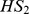	0	1,000000	18	0,999931
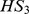	0	1,000000	12393	0,952723
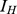	123512	0,001979	261080	0,000000

The exhaustively generated graphs with 9 vertices (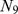) warrants special attention. In contrast to the previously introduced measures 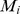, Table 3 shows that the uniqueness of 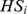 is high. More precisely, 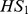 and 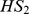 can discriminate 99% of the graph uniquely. The discrimination power of 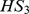 is approximately 95%. This marks a considerable improvement compared with the measures 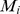 evaluated in [3] on the same classes of graphs. The improvement of the discrimination power of the new measures can be explained by the fact that partial Hosoya polynomials and graph spectra capture quite different aspects of graph structure. In particular, the partial Hosoya polynomial captures local graph properties related to distances in a graph, and the indices 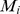 take account of the moduli of the zeros of these polynomials. By contrast, the spectrum of a graph captures connectivity properties linked to its adjacency matrix. The combination of these graph properties in the measures 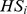 plausibly accounts for their superior performance over the single property measures (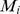).

Evidently, the discrimination power of 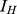 declines as the graph classes grow in size, i.e., the greater the cardinality of the graph class, the lower is index's discrimination power (measured by ndv and 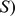, see Table 1–3. Even for small classes, the degeneracy is high. For 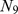, the Hosoya information content 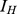 cannot discriminate at all and, hence, 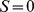. These results are not surprising in view of the definition of Hosoya information content. The blocks of the partitions consist of vertices with the same partial Hosoya polynomial. Thus, the more cycles in a graph, the greater the likelihood of obtaining large blocks of vertices with the same partial Hosoya polynomial. The occurrence of such large blocks results in high values for the quantity ndv (and low values for 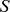).

## Summary and Conclusions

In this paper, we defined the Hosoya-Spectral indices as well as the Hosoya information content of a graph. The former measures combine structural information captured by partial Hosoya polynomials and graph spectra. It is evident that those two graph features capture structural information differently and, hence, the resulting measures may be more unique than the ones (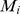) used in earlier work, see [3]. The numerical study reported here has confirmed this conjecture for both trees and graphs. Finally, as expected, the discrimination power of Hosoya information content was found to be very low.

In future research, we plan to explore extremal properties of both measures. In particular, Hosoya information content is related to the orbit structure of a graph, and this calls for studying the automorphism groups of certain classes of graphs.
